# Mercury biomonitoring in German adults using volumetric absorptive microsampling

**DOI:** 10.1007/s10661-022-09962-1

**Published:** 2022-03-30

**Authors:** Anastasia Koutsimpani-Wagner, Caroline Quartucci, James P. K. Rooney, Stephan Bose-O’Reilly, Stefan Rakete

**Affiliations:** 1 Institute and Clinic for Occupational, Social and Environmental Medicine, University Hospital, LMU Munich, Ziemssenstraße 5, 80336 Munich, Germany; 2 Institute for Occupational Health and Product Safety, Bavarian Health and Food Safety Authority, Environmental Health, Munich, Germany; 3 Academic Unit of Neurology, Trinity Biomedical Sciences Institute, Trinity College Dublin, Dublin, Ireland; 4 Department of Public HealthInstitute of Public Health, Medical Decision Making and Health Technology AssessmentMedical Informatics and Technology, Health Services Research and Health Technology Assessment, UMIT - Private University for Health Sciences, Hall i.T., Austria

**Keywords:** Mercury, Human biomonitoring, Microsampling, VAMS, Volumetric absorptive microsampling, Direct mercury analysis, Germany

## Abstract

Mercury (Hg) is a global pollutant and a danger to human health. Human biomonitoring of Hg using a dried blood matrix instead of venous blood sampling for exposure assessment is of growing interest. This study aims to develop, validate, and evaluate the application of volumetric absorptive microsampling (VAMS) for Hg biomonitoring in humans. Sampling, drying, and storage conditions were evaluated during method development. Storage in pre-cleaned glass vials after drying for 2 h in a desiccator ensured analyte stability for at least 4 weeks. Sixty-eight paired capillary VAMS and venous blood samples from volunteers in Munich, Germany, were used for method validation. Hg levels in VAMS and venous blood samples were determined by direct mercury analysis. The limits of detection and quantitation for VAMS were 0.18 and 0.61 µg/l, respectively. However, sensitivity could be improved by using two microsamples for analysis instead of one. Hg levels in VAMS samples correlated very well with Hg levels in venous blood samples (R^2^ = 0.958). Furthermore, VAMS showed a high accuracy (median recovery: 117%) and precision (median relative standard deviation: 8.7%), especially for Hg concentrations above 1.0 µg/l. In fact, accuracy and precision of VAMS improved with increasing Hg concentrations. In conclusion, VAMS in combination with direct mercury analysis is an accurate and viable alternative for human biomonitoring of Hg.

**Graphical abstract:**

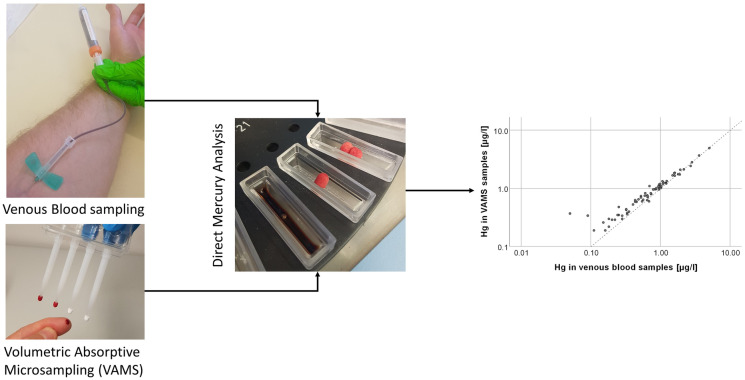

**Supplementary information:**

The online version contains supplementary material available at 10.1007/s10661-022-09962-1.

## Introduction

Mercury (Hg) is an ubiquitous trace metal toxic to humans and a global pollutant (UNEP, 2013a, b, 2019; WHO, 2008a). Major exposure pathways are the consumption of seafood (Mergler et al., 2007; Sheehan et al., 2014; WHO, 2008b), living or working in artisanal and small-scale gold mining (ASGM) communities, and working as mercury miners or in dental offices that use amalgams (Basu et al., 2018; Clarkson & Magos, 2006; Ha et al., 2017; UNEP, 2019). Moreover, Hg can be found in dental amalgam fillings, skin-whitening creams, fluorescent light bulbs, chloralkali factories, coal and other fossil fuel burning power plants, and as the preservative thiomersal (ethylmercury) in vaccines (Clarkson & Magos, 2006; Ha et al., 2017; Joy & Qureshi, 2020; WHO, 2008a).

The exposure to Hg is of great concern to human health (WHO, 2008a, b). Hg affects the nervous, cardiovascular, renal, and reproductive system, with prenatal exposure being of severe importance for the developing brain (Bose-O'Reilly et al., 2010; Clarkson & Magos, 2006; Ha et al., 2017; WHO, 2008a). For exposure assessment, Hg is usually analyzed in blood, urine, and hair (Basu et al., 2018). For the analysis of Hg in blood, venous blood sampling is the gold standard (Clarkson & Magos, 2006). However, this sampling method requires trained phlebotomists and special equipment. Furthermore, the samples need to be cooled or frozen for shipment, e.g., from the study sites to a laboratory, or for long-term storage. Consequently, venous blood sampling is associated with relatively high logistical costs for human biomonitoring, especially in remote regions like ASGM areas (Basu et al., 2018; Guerra Valero et al., 2018; Lehner et al., 2013; Ostler et al., 2014).

Microsampling methods are of growing interest in human biomonitoring as sampling, storage, and transport are relatively simple (Ostler et al., 2014). They enable a quick and minimally invasive sample collection, e.g., via finger or heel prick, of a small blood volume (10 to 100 µL) (Chapman et al., 2014). Furthermore, microsamples commonly do not require excessive storage space or cooling, thus simplifying transport of even a large number of samples (Basu et al., 2017; Demirev, 2013; Denniff & Spooner, 2014; Ostler et al., 2014). Therefore, microsampling techniques may be of great advantage to assess the exposure to environmental pollutants such as Hg in pediatric populations and especially in settings where laboratory and medical infrastructure is insufficient, such as ASGM areas (Bolea-Fernandez et al., 2016; Denniff & Spooner, 2014; Guerra Valero et al., 2018; Lehner et al., 2013).

The most cited microsampling method dried blood spots (DBS) was first described by Susi und Guthrie in the early 1960s for phenylketonuria screening in neonates (Hannon & Therrell, 2014). Since then, DBS has been widely used for numerous applications (Demirev, 2013). There are a few studies on Hg analysis with DBS showing overall good correlations, with the drawbacks of background contamination of the cards as well as the known hematocrit dependency of this method (Basu et al., 2017; Chaudhuri et al., 2009; Funk et al., 2013, 2015; Nakadi et al., 2020; Nelson et al., 2016; Nyanza et al., 2019a, b; Perkins & Basu, 2018; Santa-Rios et al., 2020a, b; Schweizer et al., 2021).

In 2014, a novel microsampling method called volumetric absorptive microsampling (VAMS) was introduced (Denniff & Spooner, 2014). Since then, more than 150 studies using VAMS for biomarker analysis in clinical and pharmaceutical settings have been published (Kok & Fillet, 2018; Protti et al., 2019). The sampling device consists of a plastic handle and a hydrophilic polymer tip. The tip absorbs a fixed volume (e.g., 23 µl) of blood by wicking. VAMS has been mainly used for the analysis of other trace metals than Hg (Anoshkina et al., 2017; Bolea-Fernandez et al., 2016; Cañabate et al., 2017; Capiau et al., 2020; Resano et al., 2018). So far, only one study has used VAMS for the analysis of Hg in certified reference material for blood (Nakadi et al., 2020). VAMS has shown to have some benefits over DBS, such as accuracy of blood volume regardless of blood hematocrit (De Kesel et al., 2015; Denniff & Spooner, 2014; Protti et al., 2019; Spooner et al., 2015). Furthermore, VAMS enables a relatively easy and ergonomic blood collection (Denniff & Spooner, 2014).

To our knowledge, VAMS not been used for human biomonitoring of Hg under field conditions. The goal of this study was the development and validation of microsampling-assisted Hg biomonitoring using VAMS in combination with direct mercury analysis. Therefore, paired VAMS and venous blood samples from non-occupationally exposed adults were analyzed for Hg.

## Experimental section

### Materials and reagents

The Hg ICP standard (1 g/l in 10% nitric acid) as well as hydrochloric acid (30%) and nitric acid (65%) for trace metal analysis were obtained from Merck (Darmstadt, Germany). Certified reference material for blood (ClinCheck^®^, 2.9 µg/l Hg) was obtained from RECIPE (Munich, Germany). Ultrapure water (resistivity > 18.2 MΩ cm) was obtained using a Milli-Q System (Merck). The VAMS Mitra™ microsampling devices (sample volume approximately 23 µl per tip) were obtained from Neoteryx LLC (Torrance, USA) and consisted of 4 VAMS tips per holder (clamshell).

Venous blood was collected into Li-Heparin-coated tubes for trace metal analysis (Sarstedt^®^) by venipuncture. For finger pricking, we used disposable lancets (Solofix) from B. Braun (Melsungen, Germany). For sample storage, plastic zip lock bags (22 × 16 cm) from Buerckle (Bad Bellingen, Germany) and 1.5 mL glass vial for chromatography (11.6 × 32 mm) with plastic screw caps from Macherey–Nagel (MN, Dueren, Germany) were used. For the cleaning of the glass vials prior to their use, the vials were washed with an aqueous mixture of hydrochloric acid and nitric acid (5% each, v/v) for 1 h on a roll mixer. The vials and lids were then rinsed twice with ultrapure water and dried for approximately 2 h at 60 °C in an oven.

### Storage stability of Hg in VAMS

In detail, VAMS samples were stored in plastic zip bags or pre-cleaned glass vials (Figure S1) for 1, 2, or 4 weeks, respectively. Furthermore, samples were stored at −20 °C, room temperature, or 40 °C, respectively. As a blood matrix, venous blood from one volunteer (1.5 µg/l Hg) was used. Each 20 µl blood was pipetted onto three VAMS sample tips. One sample tip was left unprepared as a blank sample. The sampling device was then left in its clamshell to dry for 30 min at room temperature. Each experiment was carried out in triplicate. For storage in glass vials, the dried tips were carefully removed from the plastic handle with an acid-washed stainless steel tweezer. Blank samples were prepared for all experiments in the same manner. For reference purposes, sample tips from three prepared VAMS sampling devices were directly analyzed after drying. Stored samples were immediately analyzed at the end of the storage time.

### Application of DBS for human biomonitoring of Hg

#### Study design

This study was reviewed by the ethics committee of the Ludwig Maximilians University of Munich (#20–091) and conducted according to The Code of Ethics of the Declaration of Helsinki for human experiments. All participants signed an informed consent form prior to the sampling and were asked to fill out a questionnaire about possible Hg exposure. From December 2020 to May 2021, 69 paired venous and capillary blood samples were collected from 65 consenting individuals of at least 18 years of age at the Clinic for Occupational, Social and Environmental Medicine, University Hospital, LMU Munich. Samples of two individuals were taken at multiple time points.

#### Sample collection

From each participant, approximately 7 mL of venous blood were collected and subsequently stored in a −20 °C freezer until analysis. For VAMS, one clamshell containing four sampling devices was used to collect capillary blood from the same participant after finger pricking. Briefly, after thorough hand washing, one finger was disinfected and laterally pricked with a sterile, disposable lancet. To avoid contamination by cell debris, tissue fluid, or the disinfectant, the first drop of blood was carefully wiped away (Bond & Richards-Kortum, 2015). Blood was then collected according to the manufacturer’s instructions (Neoteryx, 2021). If the blood flow was slowing down, the pricked finger was firmly wiped with gauze. The sampling devices were subsequently removed from their clamshell and placed upright in a desiccator (Figure S2) and dried for approximately 2 h at ambient conditions. Finally, the dried tips from each participant were removed with an acid-washed stainless steel tweezer, collectively placed in a pre-cleaned glass vial, and stored for 1 week at room temperature in the dark. All finger pricks were collected by a single investigator. Blank tips of each VAMS batch (52 clamshells per batch) were analyzed for assessment of background Hg levels.

### Analytical instruments and sample analysis

All measurements were carried out using a direct Hg analyzer (DMA80-evo^®^, MLS Mikrowellen-Labor-Systeme GmbH, Leutkirch, Germany). Quartz sample boats were preconditioned daily to eliminate residual traces of Hg. Hg was detected by atomic absorption at 253.5 nm. Quantitation based on an external calibration and the mean background signal of VAMS samples was subtracted from the signal. An aqueous Hg standard solution (10 µg/l) and a certified reference material for blood (2.9 µg/l) were used for quality assurance. For each run, samples were only analyzed if the standard and the certified reference material were within the specifications (9.5–10.5 µg/l and 2.3–3.5 µg/l, respectively).

For venous blood samples, 100µL were directly pipetted into the quartz sample boats, and each sample was analyzed in triplicate. The dried VAMS sampling tips, each containing approximately 23 µl of capillary blood, were individually placed on the sample boats (sVAMS). For a fraction of the VAMS samples (*n* = 24), two instead of one sample tip were used, resulting in a total analyzed blood volume of 46 µl (dVAMS). The limits of detection (LOD) were 0.02 µg/l for venous blood, 0.18 µg/l for sVAMS, and 0.10 µg/l for dVAMS, respectively. The limits of quantitation (LOQ) were 0.04 µg/l for venous blood, 0.61 µg/l for sVAMS, and 0.33 µg/l for dVAMS, respectively. Blank VAMS sample tips from the storage experiment and from each batch were analyzed in order to assess Hg background levels and possible contamination and for LOD/LOQ calculation (Table S1).

### Statistical analysis

Excel 2016 was used for processing of the data, while statistical analysis was carried out with IBM SPSS® Statistics, version 26, and R statistical software, version 4.05. The data of one participant with Hg levels in venous blood below the LOQ were excluded from statistical analysis. Consequently, the data of 68 paired samples from 64 participants were used for statistical analysis. The recovery as a measure of accuracy was calculated from the Hg levels of both methods ($recovery=\frac{{Hg}_{VAMS}}{{Hg}_{VB}}*100 \%)$. Data was graphically displayed using bar charts and scatter plots, and a Bland–Altman plot was used to graphically assess for bias between the methods. Descriptive statistics of the relative standard deviation, recovery, and number of samples below the LOD and LOQ stratified by Hg levels in venous blood (< 0.5 μg/l, 0.5–1.0 μg/l, 1.0–1.5 μg/l, > 1.5 μg/l) were calculated. Finally, a loess regression was used to visualize the recovery percentage versus venous blood percentage.

## Results and discussion

### Storage stability of Hg in VAMS

**Fig. 1:**
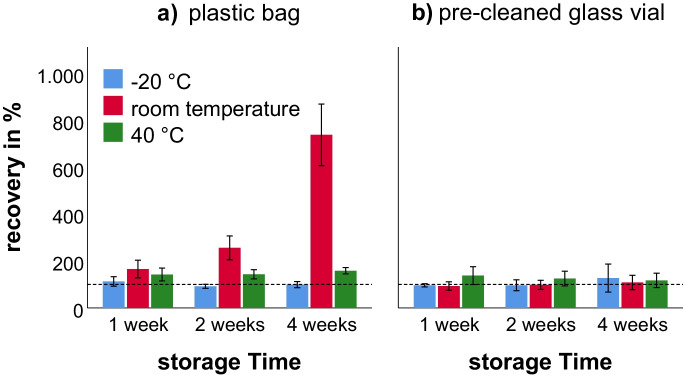
Effect of storage conditions (time, temperature) on the Hg recovery in VAMS samples stored in plastic bags (**a**) or pre-cleaned glass vials (**b**). Each bar represents mean values of the individual analysis of nine VAMS sample tips. The standard deviation is given as error bars. The dotted line represents a recovery of 100%

The first objective of the study was the investigation of different storage conditions and their effect on Hg recovery. Therefore, VAMS samples were stored in different vessels (plastic bags vs pre-cleaned glass tubes) and at different temperatures (−20 °C vs room temperature vs 40 °C) for 1, 2, and 4 weeks, respectively. In Fig. 1, mean recoveries of Hg in VAMS under different storage conditions are shown. For plastic bags (a), recoveries between 88 and 708% were observed. For pre-cleaned glass vials (b), recoveries were between 91 and 135%. When stored at room temperature in plastic bags, Hg recoveries increased with the storage time to 165%, 256%, and 708% after 1, 2, and 4 weeks, respectively. However, this effect was not observed at −20 °C (113% vs 92% vs 99%) and less prominent at 40 °C (142% vs 143% vs 158%), respectively. The increase of recovery for samples stored in plastic bags may be explained by the contamination either from the plastic bag itself or from Hg present in ambient air that penetrates through the bag. This phenomenon has also been observed in a previous study with DBS samples (Schweizer et al., 2021). In contrast, temperature (107% at −20 °C vs 100% at room temperature vs 127% at 40 °C) and time (109% at 1 week vs 107% at 2 weeks vs 118% at 4 weeks) had little effect on Hg recovery when the samples were stored in pre-cleaned glass vials. Consequently, they were used for VAMS storage during the field validation study. The only other study that has used VAMS for Hg analysis used reference material instead of fresh human blood and did not explore analyte stability or storage conditions (Nakadi et al., 2020). Blanks were prepared in the same manner but showed no differences in background Hg levels between any storage conditions.

### Correlation between venous blood and VAMS samples

The validation study with 65 individual participants aimed to explore differences of Hg concentrations in venous blood versus capillary blood sampled with VAMS. For some participants, only three full sample tips were used for instrumental analysis due to insufficient blood volume or potential contamination (e.g., accidental touching of the sample tip with the finger or another surface). The Hg level in one venous blood sample was below the LOQ, and the participant was therefore excluded from statistical analysis. From two participants, blood samples were collected at multiple times. In total, 68 paired capillary VAMS and venous blood samples were analyzed for method validation. Selected results from venous blood and VAMS samples are shown in Table 1. Additional stratification of Hg levels in venous blood by gender, age, fish consumption, and dental amalgam fillings and individual paired mean values in venous blood and VAMS samples can be found in the supplementary material (Tables S2 and S3). In summary, Hg levels in the study population showed a non-parametric distribution and were comparable to what has been previously published for German and US adults (CDC, 2019; Wilhelm et al., 2004).

**Table 1:** Hg levels in venous blood and VAMS samples (*n* = 68)

	**GM µg/l**	**Median µg/l**	**Min µg/l**	**Max µg/l**	**Min–mean–max RSD in %**	**Samples (%) < 20% RSD**
**Venous blood**	0.62	0.66	0.05	4.99	0.2–3.4–18.5	68 (100%)
**VAMS**	0.78	0.78	0.19	4.95	0.1–8.7–38.7	62 (91%)

The precision of the analysis of venous blood samples was superior to VAMS samples. All venous blood samples were below a relative standard deviation (RSD) of 20%. In contrast, 91% of VAMS showed a RSD lower than 20%, with a higher mean RSD compared to venous blood samples (8.7% vs 3.4%). This can be explained by the relatively low blood volume in VAMS samples (23 µl vs 100 µl for venous blood) and therefore a lower absolute amount of Hg per analysis, which results in a higher variation between replicates. In fact, RSD improves with higher Hg levels (Table 2). The precision of VAMS sampling was comparable to that of Hg analysis using dried blood spot (DBS) sampling previously published by the authors (93% < 20% RSD, mean RSD = 8.2%) (Schweizer et al., 2021).

**Table 2:** Individual VAMS recovery data for different Hg concentration ranges

**Hg level in venous blood**	** < 0.5 µg/l**		**0.5–1.0 µg/l**		**1.0–1.5 µg/l**	** > 1.5 µg/l**
Type of analysis	sVAMS	dVAMS	sVAMS	dVAMS	sVAMS	sVAMS
Sample number	14	10	10	14	10	10
Mean RSD^a^	15.8	7.4	9.6	4.3	8.3	5.6
Samples < LOQ^b^	13 (93%)	3 (30%)	0 (0%)	0 (0%)	0 (0%)	0 (0%)
Samples with 70–130% recovery	5 (36%)	3 (30%)	8 (80%)	12 (86%)	10 (100%)	10 (100%)
Median recovery (%)	134	140	116	115	108	103
Min. recovery (%)	108	107	89	98	92	91
Max. recovery (%)	740	194	158	142	126	117

^a^*RSD* relative standard deviation

^b^*LOQ* limit of quantitation, sVAMS: 0.61 µg/l, dVAMS: 0.33 µg/l

**Fig. 2:**
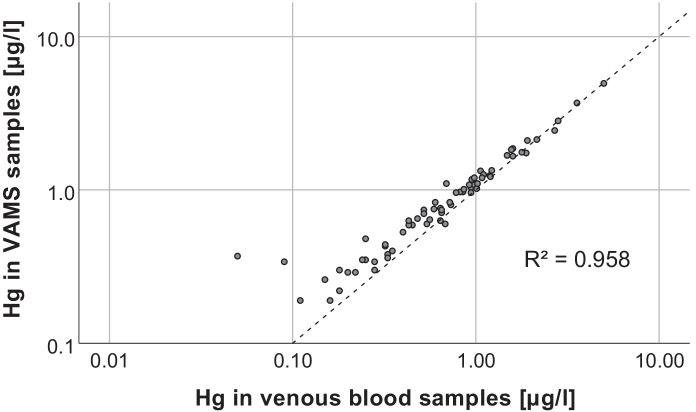
Scatter plot of Hg levels in venous blood vs Hg levels in corresponding VAMS samples. The dotted line represents the identity line

**Fig. 3:**
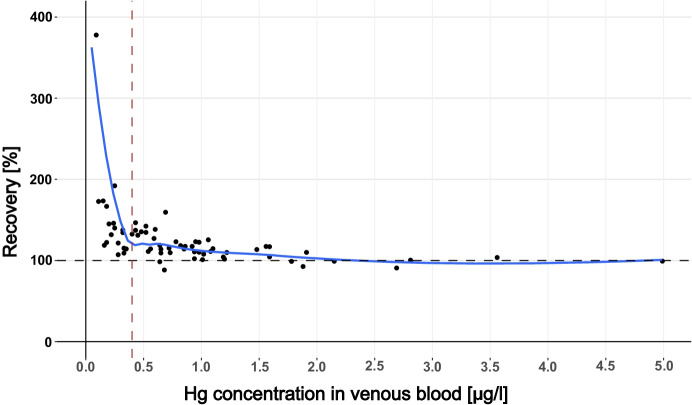
Plot of recovery vs Hg concentration in venous blood with a loess regression fit. The horizontal dashed black line represents a recovery of 100%. The vertical dashed redline represents an Hg concentration in venous blood of 0.4 μg/l

**Fig. 4:**
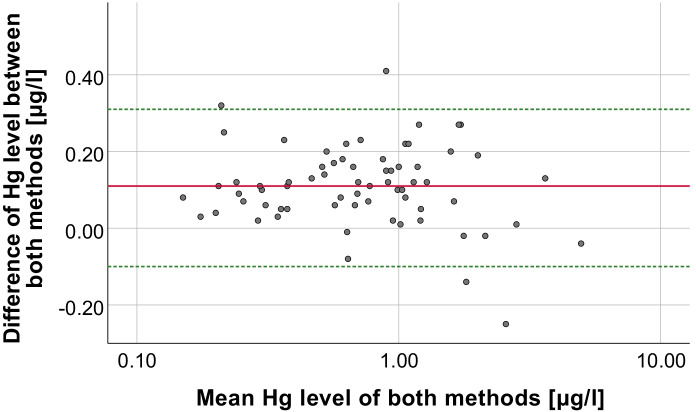
Bland–Altman plot of the absolute differences between Hg levels in venous blood and VAMS samples vs the mean of Hg levels in both samples. The mean difference (bias, solid red line) was at 0.11 μg/l. The upper and lower statistical limits (−0.10 μg/l, 0.31 μg/l, dashed green lines) were calculated by adding 1.96 times the standard deviation of the calculated differences to the mean bias

The Hg levels in VAMS and venous blood samples showed a very strong linear relationship (Spearman-Rho, R^2^ = 0.958, *p* < 0.001), which is shown in Fig. 2. This is comparable to what has been found in previous studies that measured Hg in paired venous blood samples and microsamples (Table 3). However, results apparently deviated from the identity line below venous Hg levels of 1 µg/l. Nevertheless, recoveries were mostly in an acceptable range, even when the Hg levels in VAMS samples were below the calculated LOQ. Table 2 shows the Hg recoveries of VAMS samples stratified by Hg levels in venous blood, while Fig. 3 showed that the recovery dramatically increases below a venous blood concentration of 0.4 µg/l and that the recovery improved with increasing Hg concentrations. This can be explained by the fact that varying Hg background levels in VAMS tips and potential contamination during sampling have a larger impact at low Hg levels in blood (< 1 µg/l) than in samples with relatively high Hg levels (> 1 µg/l). Overall, Hg levels in VAMS samples were on average 0.11 µg/l higher compared to the Hg levels in the corresponding venous blood samples (bias) as can be seen in the Bland–Altman plot (Fig. 4). Nevertheless, 65 of 68 samples were within the 95% confidence interval (−0.1 to 0.31 µg/l), demonstrating the good agreement between both methods. The bias is not significantly affected by Hg levels in blood, which explains why VAMS samples with high Hg levels showed the best recoveries. Besides individual Hg background levels and potential contamination, the bias may also be explained by variation in the wicking volume or differences in Hg levels between capillary and venous blood (Enderle et al., 2016; McDade et al., 2007).

**Table 3:** Summary of human biomonitoring studies that analyzed Hg in paired venous blood samples and microsamples

	**Matrix**	**Analyte**	**Hg analysis**	***n***	**Limit of detection**	**Correlation (R**^**2**^**)**
This study	VAMS	Total Hg	DMA^a^	68	0.09 µg/l	0.958
Nyanza et al. (2019)	DBS	Total Hg	ICP-MS	44	0.012 µg/l	0.976
Santa-Rios et al. (2020a)	DBS	Methylmercury	GC-CVAFS	49	0.3 µg/l	0.80
Schweizer et al. (2021)	DBS	Total Hg	DMA	44	0.14 µg/l	0.90

^a^Direct mercury analysis

For a selected number of participants with Hg levels in venous blood < 1 µg/l (*n* = 24), two VAMS tips instead of one were simultaneously analyzed in order to increase the sensitivity by simply increasing the analyzed blood volume, resulting in two instead of four replicate analysis per participant (dVAMS). Interestingly, this did not result in the improvement of the median recovery nor in the percentage of samples with a recovery between 70 and 130% (Table 2 and Fig. S3). However, the precision of the analysis improved when using dVAMS. Therefore, the use dVAMS seems advantageous for future studies in order to achieve better sensitivity and precision.

### Strengths and limitations of the study

The strength of this study is the analysis of Hg in paired capillary VAMS and venous blood samples from humans, and this is to our knowledge the first study that evaluates this novel sampling methodology in comparison to the gold standard of venous blood sampling. Additionally, the combination with direct Hg analysis enables a simple sample analysis as it does not require any sample preparation such as extraction for ICP-MS analysis (Capiau et al., 2020). Furthermore, another key element of our study is the investigation of storage conditions at multiple temperatures and in different vessels using venous blood for method development instead of certified reference materials.

A limitation of our study is that about 24% of the analyzed VAMS samples were below the calculated LOQ. This was mainly the case for relatively low Hg concentrations (< 0.5 µg/l) and for single VAMS analysis (sVAMS). This is due to the low blood volume sampled by a VAMS tip (23 µL). However, we showed that the simultaneous analysis of two VAMS tips (dVAMS) improved the sensitivity. The sensitivity may be further improved by using other analytical techniques such as ICP-MS or larger sampling tips (e.g., 30 µl). Furthermore, capillary blood consists not only of venous blood but also arterial blood and interstitial fluid (Enderle et al., 2016), and, therefore, results may not be identical (McDade et al., 2007). This could lead to variable results for Hg, since methylmercury is mainly bound to the erythrocytes, while inorganic Hg is distributed between plasma and erythrocytes (Sheehan et al., 2014). However, in recent studies, no significant differences between capillary and venous blood were found for methylmercury and total Hg (Santa-Rios et al., 2020b; Schweizer et al., 2021).

## Conclusion

This study evaluated the suitability of VAMS as an alternative blood sampling method for Hg biomonitoring of the general population. To our knowledge, this is the first study that successfully analyzed Hg in paired venous blood and VAMS samples. In conclusion, we were able to demonstrate that this sampling method produces reliable and accurate results and can therefore be used for Hg exposure assessment in studies with human subjects with limitations for low-exposed individuals (Hg levels < 0.4 µg/l).

## Supplementary information

Below is the link to the electronic supplementary material.

Supplementary file1 (PDF 405 KB)

## Data Availability

Individual anonymized Hg levels of the participants can be found in the supplementary material. Further anonymized data may be made available upon reasonable request.
