# Supplementary material for: Mercury biomonitoring in German adults using volumetric absorptive microsampling
Source: Environ Monit Assess. 2022 Mar 30;194(4):315. doi: 10.1007/s10661-022-09962-1 (PMC8969040; doi:10.1007/s10661-022-09962-1)

Supplementary Material

Table S1: Blank signals for VAMS sample tips, mean and standard deviation of blank signals used for the calculation of the limits of detection (LOD) and quantitation (LOQ). Absolute Hg levels were calculated by using the external calibration. For sample analysis, the mean blank signal was subtracted from the sample's signal. The sample volume for sVAMS (single VAMS tip analysis) was 23 µl, for dVAMS (double VAMS tip analysis) 46 µl. LOD and LOQ were calculated as follows:

$$LOD\ [\mu g/l] = \frac{3 * SD_{signal}\ [ng]}{V_{VAMS}\ [\mu l] * 1000} \qquad LOQ\ [\mu g/l] = \frac{10 * SD_{signal}\ [ng]}{V_{VAMS}\ [\mu l] * 1000}$$

	blank signals	
	**sVAMS**	**dVAMS**
	0.0002	0.0005
	0.0003	0.0005
	0.0006	0.0007
	0.0006	0.0007
	0.0006	0.0007
	0.0007	0.0008
	0.0007	0.0009
	0.0008	0.001
	0.0008	0.0011
	0.0008	0.0011
mean (signal)	0.00061	0.00080
SD (signal)	0.00020	0.00021
SD (Hg in ng)	0.00141	0.00150
LOD (µg/l)	0.18	0.10
LOQ (µg/l)	0.61	0.33

Table S2: Hg levels in venous blood samples stratified by gender, age, fish consumption and dental amalgam. All Hg levels are given in µg/l. Multiple samplings of the same participants were excluded.

		n	GM	Min	P25	Median	P75	P95	Max
All		64	0.62	0.05	0.32	0.65	1.1	2.78	4.99
**Gender**	**male**	25	0.57	0.11	0.33	0.69	0.98	2.41	2.69
	**female**	39	0.62	0.05	0.32	0.64	1.19	3.56	4.99
**Age (years)**	**18-30**	24	0.55	0.05	0.31	0.66	0.99	3.14	3.56
	**30-45**	31	0.67	0.16	0.32	0.65	1.19	3.68	4.99
	**45-60**	8	0.53	0.15	0.29	0.63	1.06	-	1.10
	**> 60**	1	0.33	-	-	-	-	-	-
**Fish consumption**	**none**	4	0.15	0.05	0.08	0.18	0.30	-	0.33
	**max 1 x / month**	18	0.38	0.09	0.18	0.34	0.60	-	1.56
	**2-4 x / month**	37	0.83	0.22	0.53	0.92	1.20	3.70	4.99
	**> 1 x week**	5	1.17	0.65	0.72	0.95	2.20	-	2.81
**Dental amalgam fillings**	**yes**	13	0.77	0.15	0.43	0.95	1.07	-	4.99
	**no**	50	0.57	0.05	0.31	0.62	1.06	2.74	3.56
	**unknown**	1	0.20	-	-	-	-	-	-

GM: geometric mean. P25/P75/P95: $25^{th}/75^{th}/95^{th}$ percentile

Table S3: Individual Hg levels (µg/l) in venous blood and paired VAMS samples

Venous blood	Paired VAMS	Venous blood	Paired VAMS
0.05	0.37 [a]	0.68	0.60
0.09	0.34 [a]	0.69	1.10
0.11	0.19 [b]	0.72	0.83
0.15	0.26 [a]	0.73	0.80
0.16	0.19 [a]	0.78	0.96
0.18	0.30 [a]	0.82	0.97
0.18	0.22 [b]	0.85	0.97
0.20	0.29 [a]	0.86	1.01
0.22	0.29 [a]	0.92	1.08
0.24	0.35	0.94	1.04
0.25	0.48	0.94	0.96
0.25	0.35	0.95	1.17
0.28	0.30 [b]	0.98	1.20
0.28	0.34 [a]	0.98	1.08
0.32	0.43 [a]	1.01	1.02
0.32	0.44	1.02	1.10
0.33	0.36 [a]	1.06	1.33
0.33	0.38 [a]	1.08	1.20
0.35	0.40	1.10	1.26
0.40	0.53	1.19	1.24
0.43	0.63	1.20	1.22
0.43	0.59 [a]	1.22	1.34
0.45	0.59 [a]	1.48	1.68
0.48	0.65	1.56	1.83
0.52	0.70	1.59	1.86
0.52	0.74	1.59	1.66
0.54	0.60	1.78	1.76
0.56	0.64	1.88	1.74
0.59	0.75	1.91	2.10
0.60	0.83	2.15	2.13
0.64	0.76	2.69	2.44
0.64	0.63	2.81	2.82
0.65	0.74	3.56	3.69
0.65	0.71	4.99	4.95

[a] < 0.61 µg/l (Limit of quantitation (LOQ) sVAMS)

[b] < 0.33 µg/l (LOQ dVAMS)

Figure S1: Storage of VAMS in plastic zip bags (left) and glass vials (right).

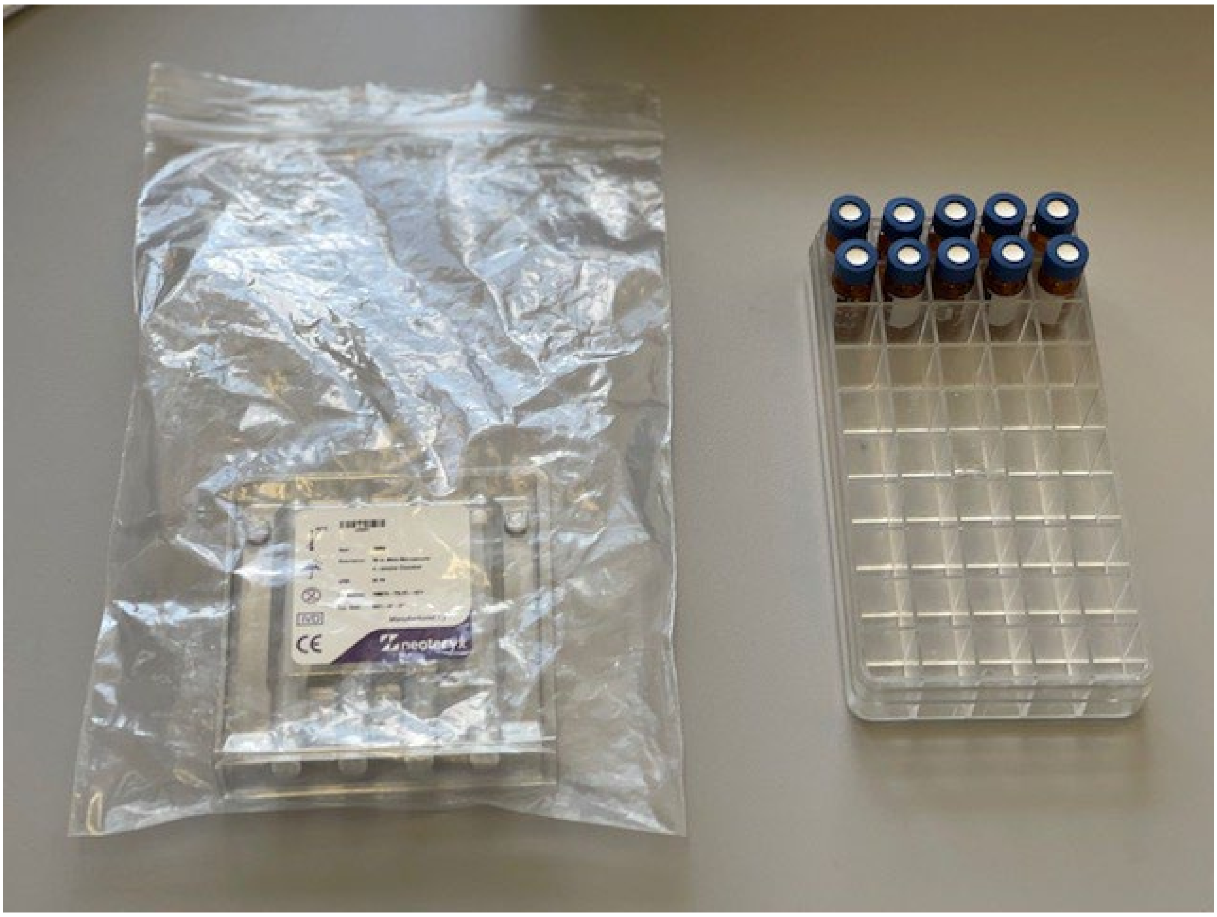

Figure S2: Drying of VAMS samples in a desiccator.

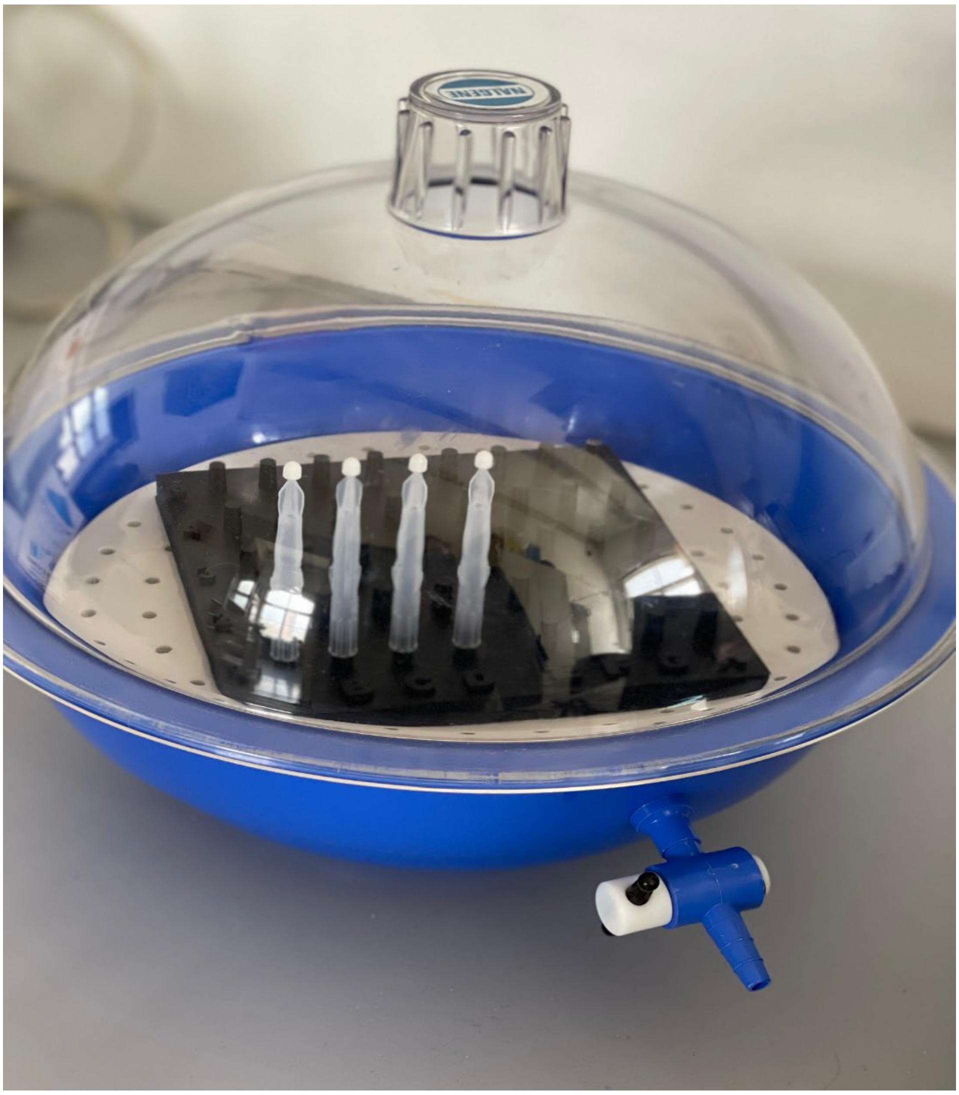

Figure S3: Recovery comparison for sVAMS (single VAMS tip analysis) and dVAMS (double VAMS tip analysis). #: Extreme values (381 % and 670 %) not shown in the graph.

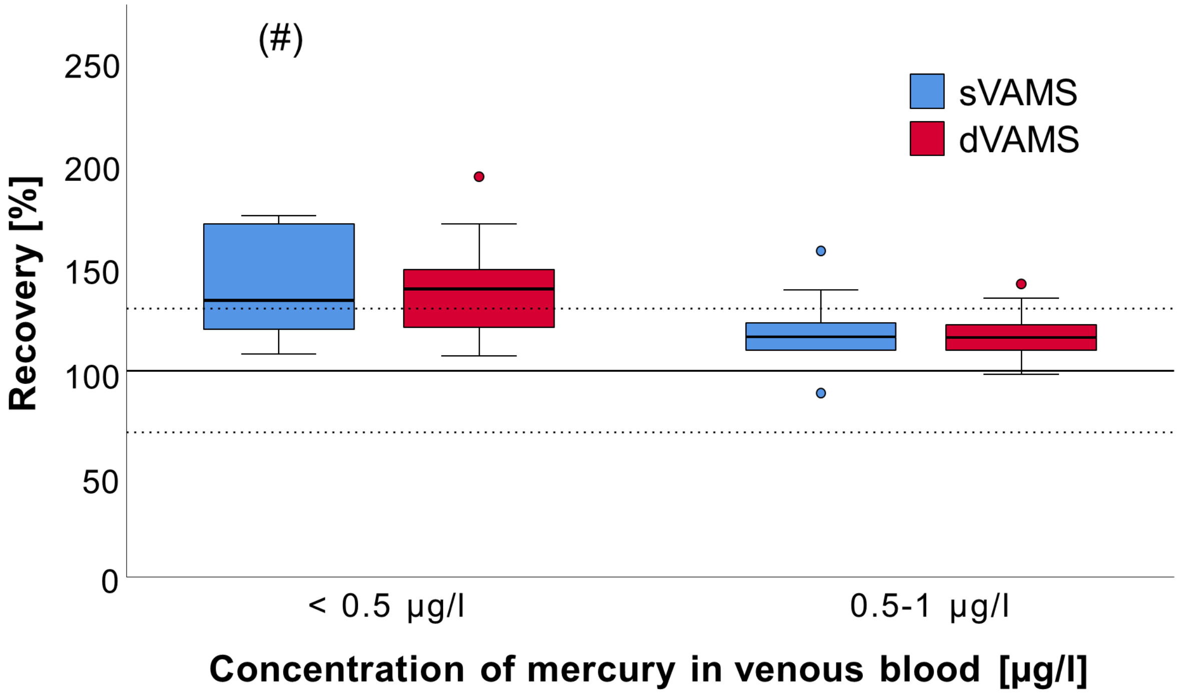

Supplement: Supplementary file 1 — Supplementary file1 (PDF 405 KB) [file 10661_2022_9962_MOESM1_ESM.pdf]
